# Influence of Thickness on the Electrical Transport Properties of Exfoliated Bi_2_Te_3_ Ultrathin Films

**DOI:** 10.1186/s11671-016-1566-7

**Published:** 2016-08-02

**Authors:** D. L. Mo, W. B. Wang, Q. Cai

**Affiliations:** 1 State Key Laboratory of Surface Physics and Department of Physics, Fudan University, Shanghai, 200433 China; 2 Collaborative Innovation Center of Advanced Microstructures, Nanjing, 210093 China

**Keywords:** Bi_2_Te_3_ ultrathin films, Exfoliation, Thickness influence, Weak antilocalization, Shubnikov de Haas oscillations

## Abstract

In this work, the mechanical exfoliation method has been utilized to fabricate Bi_2_Te_3_ ultrathin films. The thickness of the ultrathin films is revealed to be several tens of nanometers. Weak antilocalization effects and Shubnikov de Haas oscillations have been observed in the magneto-transport measurements on individual films with different thickness, and the two-dimensional surface conduction plays a dominant role. The Fermi level is found to be 81 meV above the Dirac point, and the carrier mobility can reach ~6030 cm^2^/(Vs) for the 10-nm film. When the film thickness decreases from 30 to 10 nm, the Fermi level will move 8 meV far from the bulk valence band. The coefficient α in the Hikami-Larkin-Nagaoka equation is shown to be ~0.5, manifesting that only the bottom surface of the Bi_2_Te_3_ ultrathin films takes part in transport conductions. These will pave the way for understanding thoroughly the surface transport properties of topological insulators.

## Background

As a unique class of condense matter materials, topological insulators (TIs) have attracted considerable attention these years for their potential applications in spintronics and quantum computation [1, 2]. TIs are characterized by intrinsic insulating bulk states and metallic surface states due to strong spin-orbit coupling. Theoretically, the Dirac-like surface states of TIs are protected by charge symmetry and time reversal invariance, to guarantee it non-trivial. As a result, the electron spin is locked with its momentum and the backscattering induced by nonmagnetic impurities is prohibited. These special natures of TIs bring forth exotic phenomena, such as quantum spin Hall effect and Majorana fermions appearing in vortex cores between the interface of TI and superconductor [1–4]. After HgTe/CdTe quantum wells, Bi_2_Se_3_, Bi_2_Te_3_, and Sb_2_Te_3_ as the second generation of three-dimensional TIs were proved with angle-resolved photoemission spectroscopy (ARPES) experiments to have the surface states exhibiting ideal single Dirac cone in energy band structures [5–7]. In recent years, mesoscopic quantum interference phenomena of these TI materials have been heatedly researched, such as Aharonov-Bohm oscillations, universal conductance fluctuations, weak antilocalization (WAL) effects and Shubnikov de Haas (SdH) oscillations, in which many relevant physical parameters have been obtained [8–13].

It is well-established that bismuth-telluride (Bi_2_Te_3_) is an important thermoelectric material. After confirmed as TI with very strong spin-orbit coupling, Bi_2_Te_3_ becomes a proper platform for investigating WAL effects. The current researches usually focus on Bi_2_Se_3_, which has a relatively large band gap in bulk (~0.3 eV). The Bi_2_Se_3_ and Bi_2_Te_3_ samples are commonly fabricated through chemical solution synthesis, molecular beam epitaxy, and chemical vapor deposition [10, 14, 15]. To utilize surface states of TI, the Fermi level of surface states must be near the Dirac point. The chemical nature of graphene ensures that the Fermi level is located naturally at the Dirac point, but it is not the case for TIs [1]. And there is a major hindrance for researching the exotic transport properties of TI surface states. The conducting bulk is usually more prevalent due to the existence of vacancies and impurities. Therefore, it is difficult to control and manipulate independently the conduction from the topological surface/edge states [16]. In order to suppress the bulk contributions to electrical transport and focus on the transport properties of surface states, two solutions can be employed: to manipulate the Fermi level by elemental doping/electric gating or to increase the surface-to-volume ratio. The ARPES and Hall transport experiments on Bi_2_Se_3_ showed that a small amount of Ca doping would result in insulating bulk, and the resistivity of the TI samples could be easily affected by Ca concentration [17]. It was found that the bulk conductance was suppressed by four orders of magnitude in the Cu doped Bi_2_Te_3_ films [18]. When the thickness of TI films is decreased to nanoscale or the nanostructures of TI materials are constructed, the surface-to-volume ratio of the samples will become larger. And the contributions from the topological surface conduction will dominate the transport properties [19, 20].

It is well-known that Bi_2_Te_3_ has a layered crystal structure, and the weak van der Waals interaction exists between its atomic quintuple layers [21, 22]. Therefore, Bi_2_Te_3_ can be exfoliated into ultrathin films with the thickness even down to several quintuple layers. In recent years, the transport properties of Bi_2_Te_3_ films have been studied widely. However, the explicit experimental investigations about the influence of the film thickness have not been reported on the electron transport of gapless surface states within our knowledge. The systematic explorations about thickness effects of TI thin films will be useful and compatible to device fabrication. In this work, the Bi_2_Te_3_ ultrathin films are prepared by means of mechanical exfoliation. The film thickness is manifested ranging from 10 to 200 nm by using scanning electron microscopy, atomic force microscopy, and Raman spectroscopy, as well as its relations with the size. The relevant transport parameters have been obtained from the measurements of WAL effects and SdH oscillations, and the influences of film thickness are discussed on the transport properties of gapless surface states. It is shown that there is only the bottom surface participating in the observed WAL conduction for the Bi_2_Te_3_ films as thin as 10 nm. The present results can provide a valuable insight into the applications of TIs in future electronic and spintronic devices.

## Methods

Owing to the layered crystal structure, the Bi_2_Te_3_ ultrathin films were produced by means of mechanical exfoliation from the commercial crystalline bulk Bi_2_Te_3_ with a purity of 99.99 %. After exfoliation, the obtained micro-flakes of Bi_2_Te_3_ were transferred onto a Si substrate with a 285-nm SiO_2_ layer on the surface. The morphology and thickness of Bi_2_Te_3_ ultrathin films were characterized mainly with scanning electron microscopy (SEM), atomic force microscopy (AFM), and micro-Raman spectroscopy. SEM experiments were performed in a Zeiss Sigma SEM system with Raith Elphy Plus, which functioned at 5 kV for topography observation and 20 kV for electron beam lithography. The AFM observations were carried out in air using noncontact mode, and Raman spectra were obtained with a laser excitation at 632 nm. In order to investigate the electrical transport properties, the four-terminal contacts were fabricated for a single Bi_2_Te_3_ ultrathin film on the SiO_2_/Si substrate by using electron beam lithography followed by the 5 nm/50 nm Cr/Au metal depositions with an electron beam evaporator and lift-off process. The electrical transport measurements were carried out, with the temperature ranging from 2 to 300 K and a magnetic field perpendicular to the sample plane, in a quantum design physical property measurement system under the pressure of 10 torr. The standard four-probe technique for transport measurements was adopted to eliminate the effects of contact resistance, with the two outer electrodes connected to a current source and the two inner electrodes to a voltmeter.

## Results and Discussion

In order to roughly know about the distributions of Bi_2_Te_3_ micro-flakes on the SiO_2_/Si substrate, the optical microscopy and SEM observations were performed. It is found that the thinner the micro-flake is, the darker its color will be under the optical microscope. And the size and surface morphology of the micro-flakes can be obtained in detail in the SEM images. It is shown that the micro-flakes have very smooth surfaces with the step-shaped edges, and their sizes can reach tens of micrometers, much larger than those of the Bi_2_Te_3_ nanoplates synthesized in chemical methods [10]. The precise information about the thickness of Bi_2_Te_3_ micro-flakes can be acquired in the AFM measurements. Their thickness is demonstrated varying from a few nanometers to several hundred nanometers, as shown in Table 1. It can be seen that the thickness of Bi_2_Te_3_ micro-flakes increases with the increasing size, unlike that of graphene exfoliated from graphite, in which van der Waals’s force is much smaller than that in Bi_2_Te_3_.

**Table 1:** Size and thickness of Bi_2_Te_3_ micro-flakes obtained from SEM and AFM observations

Size (μm)	1–5	5–10	10–20	20–50	>50
Thickness (nm)	10–15	15–30	30–70	70–200	>200

For bulk Bi_2_Te_3_, it is known that its crystal structure belongs to space group R‾3m (D_3d_^5^). And in one unit cell, five atomic layers can be discerned, which commonly called a quintuple [21]. In Raman spectra of Bi_2_Te_3_, the most distinct features are E_g_^2^ peak (E^2^) at ~103 cm^−1^ and A_1g_^2^ peak (A^2^) at ~133 cm^−1^. It is also reported that the intensity ratios of the E_g_^2^ peak to the A_1g_^2^ peak can be used to evaluate the thickness of Bi_2_Te_3_ films [23]. The A_1u_ peak at ~117 cm^−1^ which is not Raman active in bulk can also emerge when the thickness of Bi_2_Te_3_ film is less than 40 nm, and it will become more and more obvious with the decreasing of the film thickness due to crystal-symmetry breaking [21, 23].

**Fig. 1:**
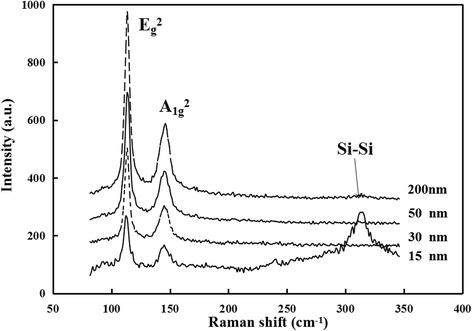
Raman spectra of the exfoliated Bi_2_Te_3_ ultrathin films with different thickness

Figure 1 is Raman spectra of the exfoliated Bi_2_Te_3_ ultrathin films with different thickness measured at room temperature. There are mainly two peaks, E^2^ at 101 cm^−1^ and A^2^ at 133 cm^−1^, excited with a 632-nm laser. The sharpness of the peaks indicates that the crystal quality of the ultrathin films is great, and the films have very few impurities and defects inside. The intensity ratios I(E^2^)/I(A^2^) for the ultrathin films at different thickness are shown in Table 2. It is displayed clearly that the ratio decreases with the decrease of the film thickness, in agreement with the previous work [23]. Because of the out-of-plane symmetry (perpendicular to the quintuple layers) no longer existing, the out-of-plane vibration mode (A^2^) can be highlighted in the Bi_2_Te_3_ ultrathin films. With the decreasing of the thickness, the E^2^ and A^2^ peaks are found in Fig. 1 slightly red-shifted, owing to the bending of the ultrathin films and the consequent strain. However, the I(E^2^)/I(A^2^) ratios in our work are much larger than those measured in the previous work [23]. It is also surprising that the A_1u_ peak at ~117 cm^−1^ cannot be observed in our Raman spectra even if the thickness of the Bi_2_Te_3_ films is as thin as 15 nm. In Fig. 1, it is distinctly exhibited that the 15-nm film is so gauzy that the Si-Si peak from the substrate (280–350 cm^−1^) can also be detected. No reasonable explanations can be given right now, although a laser light of 488 nm has been noticed to be used in the previous works [21, 23].

**Table 2:** The intensity ratios of E^2^ to A^2^ Raman peak at different film thickness

Thickness (nm)	200	50	30	15
I (E^2^)/I (A^2^)	3.33	2.84	2.67	2.50

**Fig. 2:**
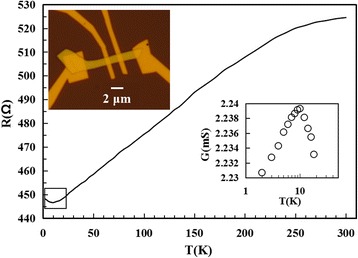
The measured resistance of the Bi_2_Te_3_ ultrathin film with a thickness of 15 nm is plotted as a function of temperature. The *left inset* shows a typical optical image of the four-terminal electrodes fabricated on a single ultrathin film. The *right inset* illustrates the details in the area marked by a square

For the investigations to electrical transport properties of the exfoliated Bi_2_Te_3_ ultrathin films, the standard four-terminal geometry devices were fabricated on the samples *via* electron beam lithography. As shown in Fig. 2, the resistance of the ultrathin film was measured as a function of temperature ranging from 2 to 300 K. A typical optical image of the exfoliated film is exhibited in the inset of Fig. 2 after the preparation of the electrodes. It can be seen that the film is almost transparent under optical microscope, on account of its extreme thinness. The right inset shows the details in the area marked by a square in Fig. 2.

Because of the tellurium vacancies or impurities existing, Bi_2_Te_3_ usually exhibits very good electrical conductivity. In Fig. 2, the resistance of Bi_2_Te_3_ ultrathin film presents principally a metallic behavior, consistent with the previous works [8, 9]. It can be seen that the resistance increases with the temperature increasing at the range of 10 to 300 K, implying that conductivity is dominated by the carrier mobility. As the temperature decreases, the phonon scattering reduces, resulting in the carrier mobility increasing and the resistance decreasing. When the temperature *T* lowers below 10 K, the resistance is found to increase with *T* dropping and appears to present *ln*T dependence below 5 K as shown in the right inset of Fig. 2, presumably due to freezing effect of the carriers, electron-electron interactions and WAL effect [24, 25]. The low temperature less than 10 K usually brings about the absence of inelastic phonon scattering [8], and impurity scattering of the charge carriers should dominate the transport [26]. This impurity scattering substantially does not change with temperature. However, the temperature dropping causes the carrier concentration to diminish, resulting in the resistance increasing. When T drops below 5 K, electron-electron interactions as well as WAL effect probably make important contributions to conduction, at last inducing the *ln*T dependence of resistance [25]. In addition, the semiconductor-like resistance below 10 K shown in Fig. 2 indicates that bulk conductance of Bi_2_Te_3_ ultrathin films is suppressed to a large extent, and surface conduction will act as a non-negligible role [24].

**Fig. 3:**
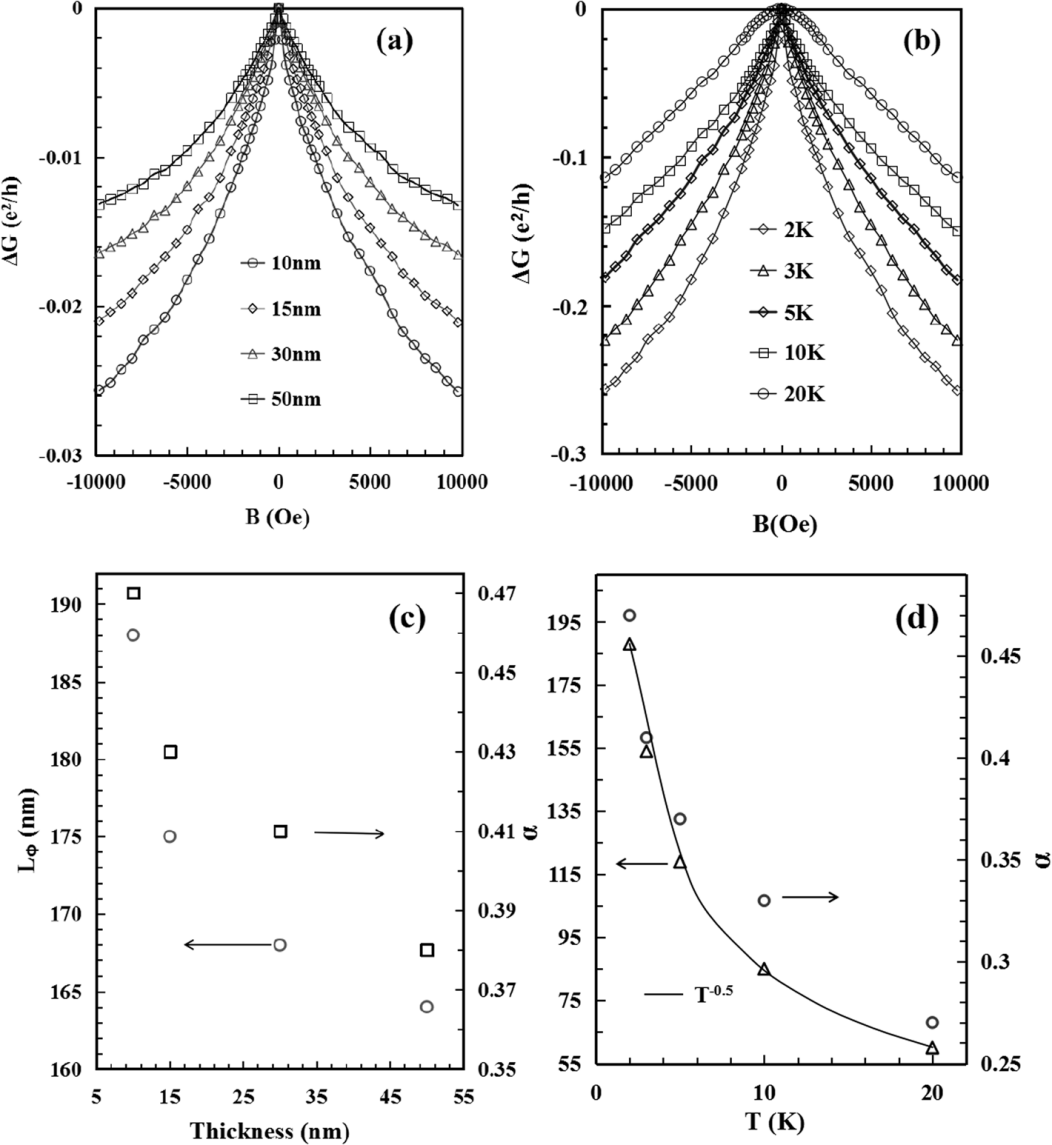
**a** The magneto-conductance obtained at 2 K on the Bi_2_Te_3_ films with different thickness. **b** The magneto-conductance curves acquired at different temperature on a 10-nm Bi_2_Te_3_ film. **c**, **d** The phase coherent length *L*_*ϕ*_ and the coefficient α are shown versus thickness and temperature, respectively. The solid line in (**d**) is the T^−1/2^ fitting of *L*_*ϕ*_

The magneto-transport properties of individual Bi_2_Te_3_ ultrathin film were investigated with the magnetic field B perpendicular to the ultrathin film. Figure 3a shows the magneto-conductance G plotted as a function of B obtained at 2 K on four samples with the thickness of 10, 15, 30, and 50 nm, respectively. Figure 3b shows the magneto-conductance acquired at different temperature on the 10-nm sample. It is clear to see in Fig. 3a, b that there are dips near *B* = 0 T, the prominent feature for the WAL effect. The WAL cusp persists to the temperature of 10 K. And at 20 K, the G-B curve exhibits a parabolic dependence at small field which is typical for metallic transport of the bulk states due to the Lorentz deflection [18]. The WAL effect can be described by a simplified Hikami-Larkin-Nagaoka (HLN) equation [27]:

1 $$ \varDelta G(B)=-\frac{\upalpha {e}^2}{\uppi h}\left[\varPsi \left(\frac{1}{2}+\frac{h}{8e\uppi {L}_{\phi}^2B}\right)- \ln \left(\frac{h}{8e\uppi {L}_{\phi}^2B}\right)\right] $$

Here, Δ*G*(*B*) = *G*(*B*) − *G*(*0*) is the change of magneto-conductance, Ψ(*x*) is the digamma function, α is a coefficient indicating the type of localization, *L*_*ϕ*_ is the phase coherent length, *h* is the Planck constant, and *e* is electronic charge. According to Fig. 3, the experimental data are fitted with the HLN equation and α = 0.47 is obtained for the Bi_2_Te_3_ ultrathin film of 10 nm at 2 K, quite close to the theoretical value of 0.5 for WAL in a single conductive channel.

The fitting parameter α is ~0.5 here, manifesting that there is only one topological surface contributing to the WAL transport for our exfoliated Bi_2_Te_3_ films. And presumably, it is the bottom surface of the ultrathin films that dominates the conduction, due to oxidation and photolithographic contaminations existing on the top surface [10]. In Fig. 3c, d, the parameters of α and *L*_*ϕ*_ extracted from the HLN fittings are plotted as the function of thickness and temperature, respectively. It is shown that α deviates from 0.5 to some extent with the increasing of film thickness, suggesting the bulk contribution to transport becoming larger and disturbing the signal from the surface states. A similar trend happens for α with the increasing of temperature. The phase coherent length *L*_*ϕ*_ is also displayed to decrease with the film thickness increased, due to the effects of the surface states on conduction lowered. *L*_*ϕ*_ can reach 188 nm for the 10-nm film. In Fig. 3d, it is noted that *L*_*ϕ*_ can be fitted well with the T^−1/2^ dependence at the temperature ranging from 2 to 20 K, indicating again that electron-electron interactions become a significant source of dephasing [28]. The T^−1/2^ dependence of *L*_*ϕ*_ is a typical characteristic of two-dimensional electron interference [18], and it gives evidence of the electrical transport through topological surface states existing in the 10-nm Bi_2_Te_3_ ultrathin film.

**Fig. 4:**
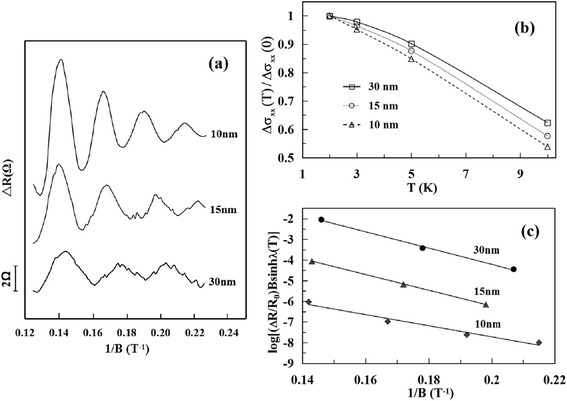
**a** The SdH oscillations obtained at 2 K on the Bi_2_Te_3_ films with thickness of 10, 15, and 30 nm, respectively. **b** The corresponding conductivity amplitudes are plotted as the function of temperature. **c** The Dingle plots at 2 K of the films with different thickness

When a material with high carrier mobility stays in a magnetic field B, the SdH oscillations will be usually observed, in which the magneto-resistance R varies periodically with 1/B due to consecutive emptying of Landau levels with the increasing of the magnetic field [29, 30]. From the R-B curves obtained experimentally at 2 K with the background subtracted, the SdH oscillations are definitely shown in Fig. 4a for the Bi_2_Te_3_ ultrathin films with different thickness. It can be found that the oscillation amplitude decreases with the increasing of thickness, implying that the surface conduction plays a major role in the SdH oscillations for the ultrathin film. And then, the oscillations are molested by the bulk transport of the thick film, where the universal conductance fluctuations come to be more pronounced. By using fast Fourier transform, the oscillation frequency will be achieved as *f*_SdH_ = 41.2 T for the 10-nm film. Since the period of SdH oscillations Δ(1/B) = 4πe/(hk_F_^2^) [8], the frequency *f*_SdH_ = hk_F_^2^/(4πe) and the extremal cross-sectional area of the Fermi surface S_F_ = πk_F_^2^, then the Fermi vector k_F_ is obtained as 0.35/nm. The cyclotron mass can be determined according to the T dependence of the amplitude Δσ_*xx*_ of conductivity oscillations: Δσ_*xx*_(T) = Δσ_*xx*_(0) λ(T)/*sinh*λ(T). For the surface states, the thermal factor λ(T) is given by

2 $$ \lambda \left(\mathrm{T}\right)\kern0.5em =\kern0.5em 2{\uppi}^2{k}_BT{m}_{cyc}/\left(\hbar eB\right) $$

Here *k*_*B*_ is Boltzmann’s constant and *m*_cyc_ is the cyclotron mass [31–33]. Figure 4b shows the normalized conductivity amplitudes Δσ_*xx*_(T) plotted as the function of temperature for the ultrathin films with different thickness. It is found that *m*_cyc_ = 0.117 *m*_0_ (*m*_0_ is the electron rest mass) by fitting the T dependence of the amplitude Δσ_*xx*_(T) to Eq. (2). Note that *m*_cyc_*V*_F_ = ħk_F_; therefore, the Fermi velocity *V*_F_ = ħk_F_/*m*_cyc_ = 3.47 × 10^5^ m/s and the Fermi level *E*_F_ = *m*_cyc_*V*_F_^2^ = 81 meV above the Dirac point for the 10-nm Bi_2_Te_3_ film. The lifetime τ of the surface states can be obtained by estimating the Dingle factor e^−D^, where *D* = 2π^2^E_F_/(*ħeB*τV_F_^2^) [24, 31, 33]. Because the amplitude of resistance oscillation ΔR/R_0_ ~ [λ(T)/sinhλ(T)]e^−D^ [24, 31], τ can be found from the slope in the plot of log(ΔR/R_0_)Bsinhλ(T) versus 1/B (shown in Fig. 4c). Then the carrier lifetime τ = 4.01 × 10^−13^ s, and the carrier mobility μ = eτ/*m*_cyc_ = 6030 cm^2^/(Vs) for the 10 nm film, seven times larger than the bulk mobility μ_*b*_ ~ 860 cm^2^/(Vs) [31]. For the experimental data obtained on the films with different thickness at 2 K, the parameters shown in Table 3 can be achieved.

**Table 3:** The estimated transport parameters from SdH oscillations observed at 2 K on the Bi_2_Te_3_ films with different thickness

t (nm)	*f*_SdH_ (*T*)	*k*_F_ (nm^−1^)	*m*_cyc_ (m_0_)	*V*_F_ (10^5^ ms^−1^)	*E*_F_ (meV)	*τ* (10^−13^ s)	*μ* (cm^2^V^*−*1^ s^−1^)
10	41.2	0.35	0.117	3.47	81	4.01	6030
15	35.3	0.33	0.107	3.51	77	3.61	5840
30	31.5	0.31	0.101	3.55	73	3.27	5680

For the exfoliated Bi_2_Te_3_ ultrathin films, the Fermi level is about 80 meV above the Dirac cone, consistent with the previous work [24]. With the thickness decreasing from 30 to 10 nm, the Fermi level moves 8 meV far from the Dirac point and the bulk valence band. According to the band structures of Bi_2_Te_3_ [6], it is testified that the Fermi level of our exfoliated Bi_2_Te_3_ ultrathin films shifts into the bulk gap, and the electrical transport properties are dominated by topological surface states for the Bi_2_Te_3_ films with very small thickness. Balandin et al. have explored the thickness dependence for the resistance and thermoelectric efficiency of the exfoliated Bi_2_Se_3_ and Bi_2_Te_3_ films [34, 35], and it is also revealed that the surface transport through the topological surface states will play more and more predominant roles with the film thickness decreased. According to Ref. [19], the mobility of Bi_2_Te_3_ films obtained with molecular beam epitaxy (MBE) growth is 521 cm^2^/(Vs). The mobility of our samples fabricated by means of mechanical exfoliation can reach 6030 cm^2^/(Vs), much higher than those of the samples obtained in MBE growth [28] and chemical method [24]. Probably because the samples in the previous works have a non-insulating substrate or a surface/crystal structure not so intact as those of our exfoliated samples. In this work, the experimental mobility of carriers is found in the range of 5680 to 6030 cm^2^/(Vs), increasing with the thickness decreased and diminishing with the bulk transport involved. It is proposed that ultra-small thickness for TIs is a good way to control and suppress the bulk contribution to the electrical transport.

## Conclusions

In summary, the Bi_2_Te_3_ ultrathin films with the thickness of several tens of nanometers have been fabricated by using mechanical exfoliation. According to the experimental results of SEM, AFM, and Raman Spectroscopy, the ultrathin films are found to possess excellent crystal quality as well as smooth surfaces, and their thickness increases with the increasing of size. The WAL effect and SdH oscillations have been observed in the magneto-transport investigations for the films with magnetic field perpendicular to the surface. It is verified that the two-dimensional transport through topological surface states plays a dominant role in conductance of the film as thin as 10 nm. The coefficient α in the HLN equation has a measurement of ~0.5 and suggests that only one surface channel contributes to the conduction. It is shown that the carrier mobility can reach ~6000 cm^2^/(Vs) for the thinner film, almost one order of magnitude larger than the bulk mobility. Ultra-small thickness is demonstrated an effective way for TIs to control and suppress the bulk contribution to transport.
